# Supplementary material for: Comparing descriptive and theoretical models of decision-making under uncertainty and their relation to socioeconomic factors
Source: PLoS One. 2025 Sep 15;20(9):e0332189. doi: 10.1371/journal.pone.0332189 (PMC12435726; doi:10.1371/journal.pone.0332189)
Supplement: S1 Appendix — This document includes methods and results for the self-report informed measurement models of decision-making under uncertainty. Moreover, we provide regression results with ADI and annual household income as the sole predictors of risk and ambiguity. Lastly, we present a proof that reparametrizes the linear subjective value model as a random intercepts and slopes linear regression. (DOCX) [file pone.0332189.s001.docx]

**SUPPORTING INFORMATION (SI) Appendix**

**SI METHODS**

*Self-report informed models*

Models incorporating self-report data (e.g., asking the participant to rate their likelihood of winning) and decision data (e.g., willingness to pay for a lottery) can improve parameter estimates by providing additional information through self-report (73,74). In the financial decision-making task, we asked participants about their perceived likelihood of winning after they reported their willingness to pay. We test if the addition of this self-report data can improve the measurement models presented in the main text. Incorporating self-report with choice data allows us to test an additional model for the risk and ambiguity parameters, in which the self-reported likelihood of winning is modeled as an additional indicator of their perceived likelihood of winning.

The self-report-informed descriptive model builds on each model specified above, in which the perceived likelihood of winning has an additional indicator:

$Self-reported likelihood of winning=(\beta_{Red Chips}+ \beta_{Grey Chips})$

Similarly, the perceived likelihood of winning in the theoretical models has a self-reported likelihood of winning as an additional indicator:

$Self-reported likelihood of winning=(p-\lambda\frac{A}{2})$

Since $(p-\lambda\frac{A}{2})$ represents a person’s perceived likelihood of winning, the self-reported likelihood of winning serves as an additional source of information for estimating $\lambda$. In this self-report-informed model, WTP still varies as a function of the equation from the linear subjective value model $\mathrm{WTP}=(p-\lambda\frac{A}{2})(R^{\alpha}$) (11). However, the perceived likelihood of winning is also determined by the self-reported probability of winning.

SI RESULTS

We used the self-reported likelihood of winning as an additional indicator of the perceived probability in both the maximal descriptive model and the theoretical linear subjective value model. We used these two models because they had the lowest LOOIC, and we wanted to test if the self-reported likelihood of winning would further improve the out-of-sample prediction. The LOOIC indicated that these models did not perform better than models without self-report data presented in the main text (see SI Table 1).

*SI Table 1: Model Comparison Results*

| **Model Name** | **Leave One Out Information Criterion (LOOIC)** | **Bayesian R^2^** |
| --- | --- | --- |
| Self-Report Informed Maximal Descriptive Model | 15858.1 (111.7) | 0.62 |
| Self-Report Informed Linear Subjective Value Model | 32847.7 (142.4) | 0.44 |

*Understanding the effects of annual household income and ADI*

To better understand the independent effect of annual household income and ADI on risk and ambiguity, we estimated separate regression models in which annual household income and ADI were included separately as predictor variables. In doing so, we learn how much the unique effect (i.e., the portion of variance explained by one variable in a multiple regression) changes when the other variable is removed. Results were consistent with the main text: annual household income remained associated with ambiguity, regardless of whether ADI is in the model or not. Further, ADI had a weak relationship with risk propensity and ambiguity aversion regardless of whether household income was present or not.

*SI Table 2: Regression Results with ADI as the sole predictor of risk and ambiguity*

| **Predictor - Outcome** | **B (89% HDI)** | **ROPE %** |
| --- | --- | --- |
| ADI – Risk Propensity | -1.03 ( -3.02, 0.96) | 21.3% |
| ADI – Ambiguity Aversion | 0.18 (-0.68, 1.02) | 62.03% |
| ADI – Risk-Ambiguity Interaction | -3.11 (-6.63, 0.44) | 6.46% |

*SI Table 3: Regression Results with annual household income as the sole predictor of risk and ambiguity*

| **Predictor - Outcome** | **B (89% HDI)** | **ROPE %** |
| --- | --- | --- |
| Annual Household Income – Risk Propensity | 0.71 (-0.95, 2.38) | 29.25% |
| Annual Household Income – Ambiguity Aversion | -0.69 (-1.37, -0.01) | 28.93% |
| Annual Household Income – Risk-Ambiguity Interaction | 2.48 (-0.66, 5.64) | 8.92% |

*Reparameterization of the linear subjective value model to the random intercepts and slopes linear regression*

We show that the linear subjective value model (11) is a special case of the maximal descriptive model when the estimate for the interaction term is 0. This proof demonstrates the relationship between the descriptive and theoretical models. They differ slightly in how one interprets the parameters of each model, but they are statistically similar.

$\boldsymbol{R}$ = number of red chips (represents winning)

$\boldsymbol{G}$ = number of grey chips (represents ambiguous information)

$\boldsymbol{p}$ = stated probability = R/100

$\boldsymbol{A}$ = Ambiguity amount = A/100

$\boldsymbol{V}$ = reward amount (constant)

Equation 1 is the expected WTP according to the linear subjective value model.

$\boldsymbol{E}\left[ \boldsymbol{WTP} \right]\boldsymbol{=}\left( \boldsymbol{p-}\boldsymbol{\lambda}\frac{\boldsymbol{A}}{\boldsymbol{2}} \right)\boldsymbol{V}^{\boldsymbol{\alpha}}$ (1)

$\boldsymbol{=}\underset{\boldsymbol{adjusted probability}}{\underbrace{\left( \frac{\boldsymbol{R}}{\boldsymbol{100}}\boldsymbol{-\lambda}\frac{\boldsymbol{G}}{\boldsymbol{200}} \right)}}\boldsymbol{V}^{\boldsymbol{\alpha}}$ (2)

$\boldsymbol{=}\frac{\boldsymbol{V}^{\boldsymbol{\alpha}}}{\boldsymbol{100}}\boldsymbol{R+}\frac{\boldsymbol{-\lambda}\boldsymbol{V}^{\boldsymbol{\alpha}}}{\boldsymbol{200}}\boldsymbol{G}$ (3)

Equation 4 is the expected WTP according to the maximal descriptive model.

$\boldsymbol{E}\left[ \boldsymbol{WTP} \right]\boldsymbol{=}\boldsymbol{\beta}_{\boldsymbol{0}}\boldsymbol{+}\boldsymbol{\beta}_{\boldsymbol{R}}\boldsymbol{R+}\boldsymbol{\beta}_{\boldsymbol{G}}\boldsymbol{G}$ + $\boldsymbol{\beta}_{\boldsymbol{RG}}\boldsymbol{R*G}$ (4)

Let β_0_ = 0 and let β_RG_ = 0:

$\boldsymbol{=0+}\frac{\boldsymbol{V}^{\boldsymbol{\alpha}}}{\boldsymbol{100}}\boldsymbol{R+}\frac{\boldsymbol{-}\boldsymbol{\lambda}\boldsymbol{V}^{\boldsymbol{\alpha}}}{\boldsymbol{200}}\boldsymbol{G + 0}\boldsymbol{R*G}$ (5)

$\boldsymbol{=}\frac{\boldsymbol{V}^{\boldsymbol{\alpha}}}{\boldsymbol{100}}\boldsymbol{R+}\frac{\boldsymbol{-\lambda}\boldsymbol{V}^{\boldsymbol{\alpha}}}{\boldsymbol{200}}\boldsymbol{G}$ (6)

$\boldsymbol{\beta}_{\boldsymbol{G}}\boldsymbol{=}\frac{\boldsymbol{-}\boldsymbol{\lambda}\boldsymbol{V}^{\boldsymbol{\alpha}}}{\boldsymbol{200}}$(7)

$\boldsymbol{\beta}_{\boldsymbol{R}}\boldsymbol{=}\frac{\boldsymbol{V}^{\boldsymbol{\alpha}}}{\boldsymbol{100}}$ (8)

We see that $\boldsymbol{\beta}$ is a combination of the ambiguity aversion and risk propensity parameters, and $\boldsymbol{\beta}_{\boldsymbol{G}}$ is the utility function scaled by 100.
