# Supplementary material for: Comparing descriptive and theoretical models of decision-making under uncertainty and their relation to socioeconomic factors
Source: PLoS One. 2025 Sep 15;20(9):e0332189. doi: 10.1371/journal.pone.0332189 (PMC12435726; doi:10.1371/journal.pone.0332189)
Supplement: Attachment — Submitted filename: Response to Reviewers.docx [file pone.0332189.s003.docx]

**Editor Comments**

**1. Please ensure that your manuscript meets PLOS ONE's style requirements, including those for file naming. The PLOS ONE style templates can be found at**

[**https://journals.plos.org/plosone/s/file?id=wjVg/PLOSOne_formatting_sample_main_body.pdf**](https://journals.plos.org/plosone/s/file?id=wjVg/PLOSOne_formatting_sample_main_body.pdf) **and**

[**https://journals.plos.org/plosone/s/file?id=ba62/PLOSOne_formatting_sample_title_authors_affiliations.pdf**](https://nam12.safelinks.protection.outlook.com/?url=https%3A%2F%2Ftrack.editorialmanager.com%2FCL0%2Fhttps%3A%252F%252Fjournals.plos.org%252Fplosone%252Fs%252Ffile%253Fid%3Dba62%252FPLOSOne_formatting_sample_title_authors_affiliations.pdf%2F1%2F010f0197fbf46ef6-91e8bec6-3975-4f33-9b09-5a107246820d-000000%2FcTw4_I4u3Xo1c5YlbYhq4muX7Yq_rqh6S3dmykyi4yk%3D219&data=05%7C02%7Cbrendan.lam%40yale.edu%7C49451191efd2416ca10a08ddc0d7f45f%7Cdd8cbebb21394df8b4114e3e87abeb5c%7C0%7C0%7C638878756233545179%7CUnknown%7CTWFpbGZsb3d8eyJFbXB0eU1hcGkiOnRydWUsIlYiOiIwLjAuMDAwMCIsIlAiOiJXaW4zMiIsIkFOIjoiTWFpbCIsIldUIjoyfQ%3D%3D%7C0%7C%7C%7C&sdata=%2BBcHNh8mkSy4lSt1625r%2Bo%2FbiIJhXWSli00cV7%2F47GU%3D&reserved=0)**.**

*Authors’ response:* We reviewed the style requirements and confirm that our submission conforms to the template.

**2. Thank you for stating in your Funding Statement:**

**“This research was supported in part by grants through the American Psychological Foundation, American Psychology-Law Society, and Harry F. Guggenheim Foundation (PI: Baskin-Sommers).”**

**Please provide an amended statement that declares *all* the funding or sources of support (whether external or internal to your organization) received during this study, as detailed online in our guide for authors at** [**http://journals.plos.org/plosone/s/submit-now**](http://journals.plos.org/plosone/s/submit-now)**.  Please also include the statement “There was no additional external funding received for this study.” in your updated Funding Statement.**

**Please include your amended Funding Statement within your cover letter. We will change the online submission form on your behalf.**

*Authors’ response:* Thank you for letting us know. We included an amended funding statement in a revised cover letter and uploaded it to the resubmission portal.

Cover letter: This research was supported in part by grants through the American Psychological Foundation, American Psychology-Law Society, and Harry F. Guggenheim Foundation (PI: Baskin-Sommers). There was no additional external funding received for this study.

**3. We note that you have indicated that there are restrictions to data sharing for this study. For studies involving human research participant data or other sensitive data, we encourage authors to share de-identified or anonymized data. However, when data cannot be publicly shared for ethical reasons, we allow authors to make their data sets available upon request. For information on unacceptable data access restrictions, please see** [**http://journals.plos.org/plosone/s/data-availability#loc-unacceptable-data-access-restrictions**](http://journals.plos.org/plosone/s/data-availability#loc-unacceptable-data-access-restrictions)**.**

**Before we proceed with your manuscript, please address the following prompts:**

**a) If there are ethical or legal restrictions on sharing a de-identified data set, please explain them in detail (e.g., data contain potentially identifying or sensitive patient information, data are owned by a third-party organization, etc.) and who has imposed them (e.g., a Research Ethics Committee or Institutional Review Board, etc.). Please also provide contact information for a data access committee, ethics committee, or other institutional body to which data requests may be sent.**

**b) If there are no restrictions, please upload the minimal anonymized data set necessary to replicate your study findings to a stable, public repository and provide us with the relevant URLs, DOIs, or accession numbers. Please see** [**http://www.bmj.com/content/340/bmj.c181.long**](http://www.bmj.com/content/340/bmj.c181.long) **for guidelines on how to de-identify and prepare clinical data for publication. For a list of recommended repositories, please see** [**https://journals.plos.org/plosone/s/recommended-repositories**](https://journals.plos.org/plosone/s/recommended-repositories)**. You also have the option of uploading the data as Supporting Information files, but we would recommend depositing data directly to a data repository if possible.**

**Please update your Data Availability statement in the submission form accordingly.**

*Authors’ response:* We received permission to upload a deidentified dataset. We updated our Data Availability statement.

**4. In the online submission form you indicate that your data is not available for proprietary reasons and have provided a contact point for accessing this data. Please note that your current contact point is a co-author on this manuscript. According to our Data Policy, the contact point must not be an author on the manuscript and must be an institutional contact, ideally not an individual. Please revise your data statement to a non-author institutional point of contact, such as a data access or ethics committee, and send this to us via return email. Please also include contact information for the third party organization, and please include the full citation of where the data can be found.**

*Authors’ response:* We revised our data statement to a non-author institutional point of contact: Yale University Institutional Review Board (HRPP@yale.edu).

**5. Please include your full ethics statement in the ‘Methods’ section of your manuscript file. In your statement, please include the full name of the IRB or ethics committee who approved or waived your study, as well as whether or not you obtained informed written or verbal consent. If consent was waived for your study, please include this information in your statement as well.**

*Authors’ response:* We revised our ethics statement in the Methods section, which specifies the full name of the IRB committee that approved the study.

Page 9: All participants provided written informed consent. All procedures were completed in line with the protocol approved by the Yale University Institutional Review Board (approval number: 1408014485).

**6. We notice that your supplementary tables are included in the manuscript file. Please remove them and upload them with the file type 'Supporting Information'. Please ensure that each Supporting Information file has a legend listed in the manuscript after the references list.**

*Authors’ response:* We deleted the supplementary tables from the manuscript file. They are now solely in the ‘Supporting Information’ file. The Supporting Information file has a legend in the manuscript after the references list (page 34).

**7. If the reviewer comments include a recommendation to cite specific previously published works, please review and evaluate these publications to determine whether they are relevant and should be cited. There is no requirement to cite these works unless the editor has indicated otherwise.**

*Authors’ response:* There were no specific citation recommendations.

**8. Please review your reference list to ensure that it is complete and correct. If you have cited papers that have been retracted, please include the rationale for doing so in the manuscript text, or remove these references and replace them with relevant current references. Any changes to the reference list should be mentioned in the rebuttal letter that accompanies your revised manuscript. If you need to cite a retracted article, indicate the article’s retracted status in the References list and also include a citation and full reference for the retraction notice.**

*Authors’ response:* We reviewed our reference list for accuracy.

**9. That said, I encourage you to strengthen the connection between your empirical findings and the economic modeling of ambiguity and risk. In particular, standard economic theory often relies on the assumptions of decreasing absolute risk aversion and decreasing absolute ambiguity aversion. Your results, however, suggest that poorer individuals invest a larger share of their income in ambiguous assets or activities than wealthier individuals. This pattern appears to contradict widely accepted theoretical predictions. In this context, I would appreciate a more explicit discussion of how your findings challenge the conventional framework and what implications this has for the behavioral assumptions underpinning economic models of decision-making under uncertainty.**

*Authors’ response:* Thank you for the suggestion. We edited the introduction and the discussion to address the point. In the introduction, we detail the mixed literature on the association between ambiguity and decision-making (page 6). In the discussion, we added a section on the implications of our findings for standard economic theory and decreasing absolute ambiguity aversion.

Page 6: Research on the relationship between socioeconomic factors and ambiguity aversion has been mixed (28,29). For example, studies using hypothetical monetary gambles or hypothetical financial investments in samples of Dutch participants (with average annual household incomes of €35,256 and €45,792, respectively) (30,31) show that individuals with higher economic resources display lower ambiguity aversion (30,31) (i.e., decreasing absolute ambiguity aversion), that is, people with more wealth appear more willing to make decisions with uncertain outcomes (e.g., investing in education or the stock market) for potentially higher returns (32). However, research conducted in developing countries using ambiguous phrases, field experiments, or a two-choice decision task tends to find different patterns for the relationship between ambiguity and decision-making depending on the experimental context. One study using ambiguous phrases shows that people living in urban areas with lower economic resources were more ambiguity seeking, whereas those living in rural areas with lower economic resources were more averse to ambiguity (28,29). Importantly, though, the effects were modified by the relative level of household income, such that within lower economic resourced families, those that had more resources show more sensitivity to ambiguity. This suggests that for the poorest people, the certainty of uncertainty (33) may lead them to be less adverse to ambiguous probabilities (34). Other studies using field experiments (e.g., distribution of crops) or a two-choice decision task show that relative income level among those in developing countries did not relate to ambiguity sensitivity (28). To date, though, no studies first compared different measurement models of decision-making before testing the relationship between socioeconomic resources and decision-making under uncertainty. This means that the mixed findings within this literature could, in part, reflect a failure to accurately estimate the decision processes of risk and ambiguity when put into context of real-world resources.

Pages 25-26: Consistent with previous research (29), we find that higher ambiguity aversion is associated with higher household income, whereas those with lower household income were less averse to ambiguity. Those with fewer resources in the household may be habituated to an environment of ambiguity in their everyday lives, which is reflected in their reduced sensitivity to ambiguity within the experimental task (70,71). Among individuals with limited household resources, chronic exposure to adversity may attenuate the perceived significance of minor financial losses. This diminished sensitivity may stem from the prioritization of handling more immediate life stressors or from an internalized expectation that financial setbacks are a normative aspect of daily life (70–72). Moreover, those with lower annual household income may be more motivated to pursue rewards despite the uncertainty, given the tenuous nature of their financial circumstances (73).

Of note, the annual household income-ambiguity effect reported here is counter to the research on the decreasing absolute ambiguity aversion assumption (74–76), which posits that individuals become less ambiguity averse as their wealth increases (30,31,74,77). However, much of the previous work supporting decreasing absolute ambiguity aversion was conducted in samples with little representation of lower income participants, unlike the present study and other psychological studies (28,29) conducted in developing countries. Further, there is growing evidence that decreasing absolute ambiguity aversion is far from a settled conclusion, with the specifics of one’s socioeconomic background or other individual characteristics playing a key role in contextualizing decision-making effects (78–81). Future research can continue to test the bounds of the decreasing absolute ambiguity aversion assumption, seeking to understand in what contexts and for whom this assumption is most relevant. Future research also should investigate the mechanisms for people’s blunted ambiguity when they have a lower household income. Notably, there was a stronger relationship between annual household income and ambiguity than with ADI and ambiguity. It could be that proximal factors (e.g., struggling to pay rent) that are more closely tied to lower household income affect one’s decision-making compared to surrounding neighborhood-level factors, which are more distal.

**Comments to the Author**

**1. Is the manuscript technically sound, and do the data support the conclusions?

The manuscript must describe a technically sound piece of scientific research with data that supports the conclusions. Experiments must have been conducted rigorously, with appropriate controls, replication, and sample sizes. The conclusions must be drawn appropriately based on the data presented.**

**Reviewer #1: Yes**

**Reviewer #2: Yes**

*Authors’ response:* We thank the reviewers for noting that our work was technically sound.

**2. Has the statistical analysis been performed appropriately and rigorously?**

**Reviewer #1: Yes**

**Reviewer #2: Yes**

*Authors’ response:* We thank the reviewers for noting that our analyses were appropriate.

**3. Have the authors made all data underlying the findings in their manuscript fully available?

The** [**PLOS Data policy**](https://nam12.safelinks.protection.outlook.com/?url=http%3A%2F%2Ftrack.editorialmanager.com%2FCL0%2Fhttp%3A%252F%252Fwww.plosone.org%252Fstatic%252Fpolicies.action%2523sharing%2F1%2F010f0197fbf46ef6-91e8bec6-3975-4f33-9b09-5a107246820d-000000%2FovH0_xc29g3evimrEv1YnDIK7-NS6OstPbN0a8s7laU%3D219&data=05%7C02%7Cbrendan.lam%40yale.edu%7C49451191efd2416ca10a08ddc0d7f45f%7Cdd8cbebb21394df8b4114e3e87abeb5c%7C0%7C0%7C638878756233562379%7CUnknown%7CTWFpbGZsb3d8eyJFbXB0eU1hcGkiOnRydWUsIlYiOiIwLjAuMDAwMCIsIlAiOiJXaW4zMiIsIkFOIjoiTWFpbCIsIldUIjoyfQ%3D%3D%7C0%7C%7C%7C&sdata=SuR7lPLnwT5RSZB5bskeaI3VM1vpSZcCJqTKnks%2FSHA%3D&reserved=0) **requires authors to make all data underlying the findings described in their manuscript fully available without restriction, with rare exception (please refer to the Data Availability Statement in the manuscript PDF file). The data should be provided as part of the manuscript or its supporting information, or deposited to a public repository. For example, in addition to summary statistics, the data points behind means, medians and variance measures should be available. If there are restrictions on publicly sharing data—e.g. participant privacy or use of data from a third party—those must be specified.**

**Reviewer #1: No**

**Reviewer #2: Yes**

*Authors’ response:* We now received approval to upload a deidentified dataset to OSF. All data and code are now publicly available.

**4. Is the manuscript presented in an intelligible fashion and written in standard English?

PLOS ONE does not copyedit accepted manuscripts, so the language in submitted articles must be clear, correct, and unambiguous. Any typographical or grammatical errors should be corrected at revision, so please note any specific errors here.**

**Reviewer #1: Yes**

**Reviewer #2: Yes**

*Authors’ response:* We thank the reviewers for noting that our work was presented in an intelligible fashion.

**5. Review Comments to the Author

Please use the space provided to explain your answers to the questions above. You may also include additional comments for the author, including concerns about dual publication, research ethics, or publication ethics. (Please upload your review as an attachment if it exceeds 20,000 characters)**

**Reviewer #1: Dear Author, The manuscript makes a valuable contribution to the field and presents its findings clearly and effectively. In future author should concentrate on more innovative work rather than comparison of existing ones.**

*Authors’ response:* We thank the reviewer for noting that our manuscript makes a “valuable contribution.” We appreciate the encouragement of the reviewer to take the foundation laid by the present study and expand to more innovative models in future work. We see the present study as a necessary step in establishing a solid framework for measurement models of risk and ambiguity in this area that will serve to guide future work.

**Reviewer #2: The paper entitled “Comparing Descriptive and Theoretical Models of Decision-Making Under Uncertainty and Their Relation to Socioeconomic Factors” has a relevant research topic. The researchers performed a comparative analysis of four models for assessing decision-making under uncertainty, which differ in the mathematical form of risk and ambiguity, to determine which one best predicts willingness-to-pay behavior in a financial decision-making task. The methods were well detailed. However, I make some considerations in order to improve the reader's understanding.**

*Authors’ response:* We thank the reviewer for noting that our methods were “well detailed” and we appreciate the suggestions for refining our presentation.

**(1) Regarding the scale of the socioeconomic measure “Household Income”, is the Household Income considered monthly or annual? I suggest specifying this in the text.**

*Authors’ response:* Household income is annual. We changed the text in the main manuscript, in the figures, and in the tables.

Page 9: Participants were asked about their annual household income…”

**(2) The abstract is well structured and the research well defined. However, the authors do not mention the methodology used to perform the comparative analysis. They also do not mention the limitations of the research.**

*Authors’ response:* Following the reviewer’s suggestion, we now provide details about the methodology in the abstract

Abstract: We used Bayesian hierarchical modeling to fit each measurement model and Leave One Out Information Criteria to assess how well each model performed.

We also speak about the limitations of our research in the abstract.

Abstract: Future research should further investigate how aspects of socioeconomic background—such as childhood economic conditions and environmental unpredictability—influence decision-making. It also is important to test whether ambiguity aversion estimates, when contextualized by socioeconomic variables, can reliably predict real-world decision-making under uncertainty.

**(3) Although the introduction reviews the types of models and describes the objective of the research, the methodology used is not mentioned (which methodology was used to build the models and to compare and evaluate the models constructed).**

*Authors’ response:* We briefly mention the methodology used to build, compare, and evaluate models in the last paragraph of the introduction.

Page 7: Using a hierarchical Bayesian analysis framework, we compare and evaluate all models with the leave-one-out information criterion.

**(4) The figures and tables are in agreement. However, on pages 17 and 18, the text cites a value for LOOIC of the Theoretical Exponential Subjective Value Model, and another value is presented in Table 2. Both Table 2 and Table 4 show a higher LOOIC for the Theoretical Exponential Subjective Value Model. I suggest checking this result.**

*Authors’ response:* Thank you for pointing this out. We corrected the text on page 18 so the LOOIC of the Theoretical Exponential Subjective Value Model is now consistent with Table 2.

**(5) Page 10 has a misconfigured sentence in the last paragraph.**

*Authors’ response:* The misconfigured sentence is corrected:

Page 10: The task consisted of 45 trials, in which the number of red chips ranged from 0 (representing a 0% known chance of winning) to 49 (representing a 49% known chance of winning) across trials.

**(6) On page 14, in “Theoretical linear subjective value model” the authors state that “Higher levels of “beta” indicate greater sensitivity to ambiguity, (...)”, however, “lambda” is the ambiguity sensitivity parameter. This needs to be checked.**

*Authors’ response:* We corrected the notation, so that “beta” is now removed when talking about the ambiguity sensitivity parameter for the theoretical linear subjective value model.

**(7) On page 12, in “Baseline descriptive random intercepts model”, 0 is given in Equation (2). In Table 1, this parameter was represented differently than what was agreed in Equation (2). I suggest keeping the same representation to avoid ambiguous information.**

*Authors’ response:* Thank you for the correction. We changed the parameter in Table 1 so it is now consistent with the equation presented in Equation (2):

Table 1’s Baseline descriptive random intercepts model ${WTP}_{ij}=\gamma_{00}+b_{0j}+ \beta_{1}{Red}_{ij}+ \beta_{2j}{Grey}_{ij}+ \beta_{3j}{Grey}_{ij}\times{Red}_{ij}+\varepsilon_{j}$

Equation (2): $\beta_{0j}=\gamma_{00}+b_{0j}$

**(8) On page 16, Equations (11) and (12) are the same. Is this correct?**

*Authors’ response:* The equations are the same, except that $\mu$ in equation 11 has a different subscript. “$\mu_{\lambda}= \beta_{0}+ \beta_{1} \times annual household income{+ \beta}_{2} \times ADI$ (11)” refers to the expected value of lambda (ambiguity aversion), whereas “ $\mu_{\alpha}= \beta_{0}+ \beta_{1} \times annual household income{+ \beta}_{2} \times ADI$(12)” refers to the expected value of alpha (risk propensity).
